# Disruption of epidermal growth factor receptor signaling and cytoskeletal dynamics by mebendazole and gefitinib synergistically impairs paracrine cytokine signaling in non-small cell lung cancer and triple-negative breast cancer Cell lines

**DOI:** 10.1371/journal.pone.0338027

**Published:** 2025-12-16

**Authors:** Mohamed El-Tanani, Shakta Mani Satyam, Syed Arman Rabbani, Yahia El-Tanani, Mark Sutherland, Frezah Muhana

**Affiliations:** 1 RAK College of Pharmacy, RAK Medical and Health Sciences University, Ras Al Khaimah, United Arab Emirates; 2 Department of Pharmacology, RAK College of Medical Sciences, RAK Medical and Health Sciences University, Ras Al Khaimah, United Arab Emirates; 3 Department of Clinical Pharmacy, RAK College of Pharmacy, Ras Al Khaimah Medical and Health Sciences University, Ras Al Khaimah, United Arab Emirates; 4 Royal Cornwall Hospital Trust, NHS, Truro, United Kingdom; 5 Department School of Chemistry and Biomedical Sciences, University of Bradford, Bradford, United Kingdom; 6 Princess Sarvath Community College, Amman, Jordan; University of Michigan, EGYPT

## Abstract

**Background:**

Aberrant paracrine cytokine signaling and dysregulated signal transduction are critical drivers of tumor progression and therapeutic resistance in aggressive cancers such as non-small cell lung cancer and triple-negative breast cancer. This study aimed to explore a dual-targeting strategy using mebendazole, a repurposed anti-parasitic agent known to disrupt microtubules, in combination with gefitinib, an epidermal growth factor receptor tyrosine kinase inhibitor. The objective was to assess the combinatorial impact on cell viability and key regulatory pathways involved in inflammation, mitotic control, and nuclear transport.

**Methods:**

Human lung adenocarcinoma (A549) and triple-negative breast cancer (MDA-MB-231) cell lines were treated with mebendazole, gefitinib, or a combination of both. Cell viability in both the cell lines was investigated using the MTT assay, while transcriptional profiling was conducted exclusively in the A549 NSCLC cell line to assess cytokine and regulatory gene modulation. Quantitative reverse transcription polymerase chain reaction was performed to evaluate changes in the expression of inflammatory cytokines (interleukin-1 beta, interleukin-6, TNF-alpha, IFN-gamma) and regulatory genes (MMP-2, STAT 4, RAN, and RCC1).

**Results:**

Combined treatment with gefitinib (1 µM) and mebendazole (0.5 µM) elicited a pronounced synergistic cytotoxic response, reducing cell viability to ~8–10% in A549 and ~15% in MDA-MB-231 cells—representing an additional >50% and ~30–40% decrease, respectively, compared to the most effective single-agent treatment- gefitinib 1 µM. Gene expression analysis revealed significant downregulation of pro-inflammatory cytokines and alterations in genes involved in mitotic regulation and nuclear transport. These changes suggest impaired intracellular signaling and reduced tumor-supportive microenvironmental interactions. The dual approach disrupted both cytoskeletal architecture and receptor-mediated signal transduction, pointing to a multifaceted mechanism of action.

**Conclusions:**

The combination of mebendazole and gefitinib effectively suppresses tumor cell viability and modulates key pathways involved in cancer progression. By targeting cytoskeletal integrity and EGFR signaling, it may disrupt cytokine and tumor–microenvironment interactions, supporting further exploration as a strategy to overcome resistance in lung and breast cancers.

## 1. Introduction

Lung cancer remains the leading cause of cancer-related mortality worldwide, with non-small cell lung cancer (NSCLC) accounting for approximately 85% of all cases [1]. Frequently diagnosed at advanced stages, NSCLC presents significant therapeutic challenges due to limited treatment options and poor survival outcomes. Although the advent of epidermal growth factor receptor (EGFR) tyrosine kinase inhibitors (TKIs) has markedly improved outcomes for patients with EGFR-mutant tumors, resistance development, tumor heterogeneity, and immune escape continue to hinder long-term efficacy [2–4]. Triple-negative breast cancer (TNBC), characterized by the absence of estrogen receptor (ER), progesterone receptor (PR), and human epidermal growth factor receptor 2 (HER2), represents another aggressive and therapeutically challenging malignancy [5]. Lacking targetable receptors, TNBC is associated with rapid progression, high recurrence rates, and limited responsiveness to conventional chemotherapy [6]. Its mesenchymal phenotype and high metastatic potential further complicate therapeutic intervention and necessitate the development of novel strategies [7].

In this context, drug repurposing has gained traction as a time-efficient and cost-effective approach to identifying new cancer therapeutics [8,9]. Mebendazole (MBZ), a benzimidazole-based anti-helminthic agent, has demonstrated considerable anticancer activity across various preclinical models [10–12]. Its mechanism of action involves binding to β-tubulin, thereby inhibiting microtubule polymerization, disrupting mitotic spindle formation, and inducing mitotic arrest. Additionally, MBZ exhibits anti-angiogenic properties and can cross the blood-brain barrier, making it a favorable candidate for combination therapy with established anticancer agents [13–15]. Gefitinib, a first-generation EGFR-TKI, has transformed the treatment landscape for EGFR-mutant NSCLC patients [16]. By competitively inhibiting ATP binding at the EGFR tyrosine kinase domain, gefitinib disrupts downstream signaling pathways such as PI3K/AKT/mTOR and MAPK/ERK [17]. Despite its clinical success in selected populations, its efficacy in wild-type EGFR NSCLC and TNBC is limited, primarily due to innate and acquired resistance mechanisms, including secondary EGFR mutations (e.g., T790M), activation of bypass tracks (e.g., MET amplification), and the influence of the tumor microenvironment (TME) [18,19]. Studies have reported promising activity of MBZ in combination with EGFR inhibitors, including gefitinib, in both lung and breast cancer models [20,21]. These prior investigations provide supporting evidence for our approach and contextualize the novelty of our findings. Combining MBZ with gefitinib offers a novel and rational therapeutic strategy that exploits complementary mechanisms of action. MBZ’s microtubule-disrupting effects may enhance cellular stress responses and mitotic catastrophe, while gefitinib’s inhibition of proliferative signaling sensitizes cancer cells to apoptotic stimuli. Together, this dual-targeting approach may overcome limitations of monotherapies, amplify cytotoxic efficacy, and extend therapeutic benefit across diverse tumor subtypes [20].

Moreover, the tumor microenvironment plays a pivotal role in shaping treatment response by modulating inflammatory cytokines and immune cell recruitment. Key cytokines such as interleukin-6 (IL-6), tumor necrosis factor-alpha (TNF-α), interleukin-1 beta (IL-1β), and interferon-gamma (IFN-γ) are critical mediators of tumor progression, immune tolerance, and therapy resistance [22]. Understanding their modulation in response to treatment may reveal the immunomodulatory potential of anticancer agents. Additionally, transcriptional regulators such as matrix metalloproteinase-2 (MMP-2), signal transducer and activator of transcription 4 (STAT4), Ras-related nuclear protein (RAN), and regulator of chromosome condensation 1 (RCC1) are involved in key cellular processes including metastasis, immune activation, nucleocytoplasmic transport, and mitosis [23,24].

A growing body of preclinical and translational research further supports the relevance of redox balance, metabolic stress, immune modulation, and cytoskeletal integrity in determining cancer therapy response, as demonstrated by studies on cancer stem cell–driven resistance, antioxidant and metabolic protectants, and systemic lifestyle-mediated effects on cancer outcomes [25–33]. Together, these findings highlight that targeting multiple biological vulnerabilities—ranging from stemness and inflammation to metabolic resilience and treatment-induced toxicity—may enhance the therapeutic potential of rational drug combinations such as MBZ and gefitinib.

This study was designed with the hypothesis that mebendazole (MBZ) and gefitinib (GEF) act synergistically by simultaneously disrupting cytoskeletal integrity and epidermal growth factor receptor (EGFR)-mediated signaling, thereby impairing paracrine cytokine communication and reducing survival in aggressive cancers. We explicitly hypothesize that by targeting complementary vulnerabilities, this combination may produce stronger cytotoxicity than either agent alone. However, as this is an *in vitro* study, we present this as a mechanistic rationale and not as a confirmed therapeutic outcome. This study aims to evaluate the cytotoxic, transcriptional, and immunological effects of MBZ and gefitinib, individually and in combination, in A549 (NSCLC) and MDA-MB-231 (TNBC) cell lines.

## 2. Materials and methods

This study was conducted exclusively using established, commercially available human cancer cell lines and did not involve human participants, identifiable data, or animal subjects; therefore, ethics approval and consent to participate were not applicable. All experimental procedures adhered to institutional research policies and standard laboratory guidelines governing the use of in vitro cell culture models, and consent for publication was not required because no human data or identifiable information were generated.

### 2.1. Cell culture and reagents

The human non-small cell lung carcinoma (NSCLC) cell line A549 and the human triple-negative breast carcinoma (TNBC) cell line MDA-MB-231 (both at passage 2) were obtained from the American Type Culture Collection (ATCC). Their selection was based on their recognized clinical significance and widespread use as validated *in vitro* models for cancer pharmacological investigations [34]. Both cell lines were procured from a certified and authenticated cell repository to ensure the validity and reproducibility of the experimental outcomes. Authentication was conducted using short tandem repeat (STR) profiling to confirm the cell line identity, and they were routinely tested for mycoplasma contamination before use. The cells were cultured in Dulbecco’s Modified Eagle Medium (DMEM), which provides a rich nutrient environment for mammalian cell growth. This medium was further supplemented with 10% fetal bovine serum (FBS), which serves as a source of growth factors, hormones, and attachment proteins essential for cellular proliferation and survival. Additionally, the culture medium contained 1% penicillin-streptomycin solution to prevent bacterial contamination and maintain sterility during the entire course of the study [35,36]. The antibiotic mixture was added under aseptic conditions during media preparation.

Cells were cultured at 37°C in a humidified incubator with 5% CO₂. The cells were monitored daily under a phase-contrast microscope to assess confluency, morphology, and the presence of any contamination. The medium was replenished every 48 hours to ensure a consistent supply of nutrients and removal of waste products. Once the cells reached approximately 70% to 80% confluency, they were detached using a 0.25% trypsin-EDTA solution. Trypsin, a proteolytic enzyme, facilitates the detachment of adherent cells from the surface of the culture flask, while EDTA chelates calcium ions to enhance trypsin activity. After trypsinization, cells were neutralized with complete medium, centrifuged, and reseeded for further expansion or experimental treatment [37].

Mebendazole (MBZ), a benzimidazole-class anthelminthic drug known for its antitumor properties, and gefitinib (GEF), a selective epidermal growth factor receptor (EGFR) tyrosine kinase inhibitor, were chosen as the primary agents of interest for this study. These agents were obtained from established pharmaceutical suppliers with accompanying certificates of analysis to confirm their purity and potency. Stock solutions of both MBZ and GEF were prepared in dimethyl sulfoxide (DMSO), a widely used organic solvent that ensures efficient dissolution of lipophilic compounds. The stock concentrations were determined based on the solubility limits and were stored at −20°C in light-protected vials to preserve chemical stability. For experimental treatments, the stock solutions were diluted using complete culture medium to achieve the required working concentrations. Care was taken to ensure that the final concentration of DMSO in all experimental and control groups did not exceed 0.1% v/v. This was critical because higher concentrations of DMSO can lead to solvent-induced cytotoxicity, confounding the effects of the test compounds. The treatment medium was freshly prepared before each experiment to ensure accuracy and consistency.

### 2.2. Treatment protocols

The treatment strategy included both monotherapy and combination therapy groups to investigate the individual and synergistic effects of MBZ and GEF on cancer cell viability and gene expression. In the monotherapy experiments, A549 and MDA-MB-231 cells were treated with varying concentrations of MBZ (0.1 µM, 0.5 µM, and 1 µM) to examine dose-dependent effects [38,39]. In parallel, cells were treated with gefitinib at concentration of 0.3 µM and 1 µM, which was determined based on previously reported IC₅₀ values in similar cell models [40,41]. For the combination therapy groups, the chosen concentrations were MBZ at 0.5 µM and GEF at 1 µM. These specific concentrations were selected based on preliminary dose optimization experiments that indicated maximal synergistic cytotoxicity at these levels without inducing excessive non-specific toxicity. The cells were seeded in appropriate multi-well plates and allowed to adhere for 24 hours before drug administration. Following this equilibration period, the cells were exposed to the drug treatments for 48 hours under standard culture conditions. The control groups consisted of cells receiving culture medium containing 0.1% DMSO without any active drug, thereby serving as a solvent control to account for the vehicle effects. All experimental groups were set up in triplicate to ensure technical reproducibility. Furthermore, the entire experiment was independently repeated three times on separate occasions to validate the findings and assess inter-assay variability. This rigorous experimental design strengthens the reliability of the observed outcomes.

### 2.3. Cell viability assay

To evaluate the impact of MBZ and GEF on cell viability, the MTT assay was employed as a colorimetric method to measure mitochondrial metabolic activity. This assay is based on the ability of viable cells to reduce the yellow tetrazolium dye (MTT) to insoluble purple formazan crystals via mitochondrial dehydrogenase enzymes. After the 48-hour treatment period, 20 µL of MTT solution (5 mg/mL) was added to each well and incubated for four hours at 37°C in the dark to allow the formation of formazan crystals [42,43]. Following the incubation, the supernatant was carefully aspirated to avoid disturbing the crystals. Subsequently, 100 µL of DMSO was added to each well to dissolve the formazan, and the plates were gently agitated to ensure complete solubilization. The absorbance was measured at 570 nm using a microplate reader. The resulting optical density values directly correlated with the number of metabolically active, viable cells in each well. The percentage of cell viability was calculated relative to untreated control cells, which were considered 100% viable [44,45]. This assay provided quantitative insights into the cytotoxic potential of the individual and combined drug treatments.

### 2.4. Quantitative real-time PCR (qRT-PCR)

qRT-PCR analysis was purposely restricted to A549 cells based on a strong mechanistic and clinical rationale. Gefitinib exerts its therapeutic effects primarily in non–small cell lung cancer through modulation of the EGFR signaling cascade, downstream cytoskeletal regulators, and associated adaptive resistance pathways—biological processes that are most authentically represented in A549 cells. Thus, focusing transcriptional profiling on this lineage allowed us to interrogate gene networks that are directly aligned with gefitinib’s established mechanism of action and clinical indication. Importantly, whereas the study by Kutkut et al. [20] utilized A549 cells solely as a cytotoxicity and migration model for liposomal drug formulations, our study specifically examines gefitinib-induced transcriptional reprogramming, thereby requiring an EGFR-relevant and mechanistically coherent NSCLC model.

Following the initial viability assessment in both A549 (NSCLC) and MDA-MB-231 (TNBC) cells, restricting qRT-PCR to A549 served to preserve statistical power, minimize multiplicity-related noise, and enable focused pathway-level interpretation within the clinically appropriate context for gefitinib. Extending transcriptional analysis to MDA-MB-231, a lineage lacking EGFR-driven oncogenic dependence, would not only dilute the mechanistic specificity of the findings but also risk generating non-informative or biologically uninterpretable comparisons. We nonetheless acknowledge the absence of parallel qRT-PCR profiling in MDA-MB-231 as a limitation of the current study, warranting future investigation.

To understand the molecular changes induced by drug treatments, quantitative real-time polymerase chain reaction (qRT-PCR) was conducted to assess the expression levels of selected genes involved in inflammation, cell cycle regulation, and immune signaling. After 48 hours of treatment, total RNA was extracted from the cells using the TRIzol reagent, a phenol-guanidine-based solution that effectively lyses cells and preserves RNA integrity. The RNA isolation was performed according to the manufacturer’s protocol, which included chloroform extraction, isopropanol precipitation, and ethanol washing steps. The quantity and purity of the isolated RNA were assessed using a NanoDrop spectrophotometer by measuring the absorbance at 260 nm and 280 nm. Samples with A260/A280 ratios between 1.8 and 2.0 were considered pure and used for further analysis. Complementary DNA (cDNA) was synthesized using a high-capacity cDNA reverse transcription kit. The reaction mix included random hexamers, dNTPs, and reverse transcriptase enzymes, and the thermal cycling was carried out according to standard protocols. The synthesized cDNA served as a template for qRT-PCR amplification. Real-time PCR was conducted using SYBR Green Master Mix, which binds to double-stranded DNA and emits fluorescence upon excitation. The amplification reactions were performed in a thermal cycler equipped with a fluorescence detection system. Gene-specific primers were used to amplify the following genes: IL-1β, IL-6, TNF-α, IFN-γ, MMP-2, STAT4, RAN, RCC1, and the housekeeping gene GAPDH. The choice of these genes was based on their established roles in inflammation, immune signaling, cell proliferation, and mitotic regulation [46,47]. Amplification conditions included an initial denaturation step followed by 40 cycles of denaturation, annealing, and extension. A melting curve analysis was conducted at the end of the reaction to confirm the specificity of the amplified products. The comparative Ct (threshold cycle) method, also known as the 2^-ΔΔCt method, was employed to calculate relative gene expression levels [36,48,49]. All reactions were performed in triplicates for accuracy and reproducibility.

### 2.5. Statistical analysis

All experiments were performed using a minimum of three independent biological replicates (N = 3), and within each biological replicate, all conditions were tested in technical triplicates. This ensures robustness, reproducibility, and statistical validity of the results. All quantitative data obtained from MTT assays and qRT-PCR were expressed as the mean ± standard deviation (SD) to represent central tendency and variability. Statistical analyses were conducted to determine the significance of differences among various treatment groups. One-way analysis of variance (ANOVA) was used as the primary statistical test to evaluate differences across multiple groups. Following ANOVA, Tukey’s post hoc test was applied to perform pairwise comparisons and identify statistically significant changes between specific treatments. A p-value of less than 0.05 was considered to indicate statistical significance. All statistical calculations and graphical visualizations were performed using Statistical Package for the Social Sciences (SPSS) software (version 30), which provides an intuitive interface for conducting robust biostatistical analyses and generating publication-quality figures. The software facilitated the plotting of bar graphs, error bars, and significance notations to clearly convey the experimental findings.

## 3. Results

### 3.1. Cell survival assays in A549 cells

In the A549 non-small cell lung cancer (NSCLC) model, untreated control cells exhibited 100% viability, serving as the reference baseline. Gefitinib (0.3 μM) reduced cell viability to ~75% (p = 0.010), indicating a moderate but statistically significant cytotoxic effect at a low concentration. At 1 µM, gefitinib induced a pronounced cytotoxic response, reducing mean cell survival to approximately 20% (p < 0.001 vs control and p < 0.001 vs gefitinib 0.3 µM), confirming a steep dose-dependent inhibition of proliferation.

Mebendazole (MBZ) monotherapy demonstrated a clear dose-dependent reduction in cell survival. At 0.1 μM, viability decreased to ~83% (p = 0.007). At 0.5 μM, it dropped further to ~69% (p = 0.001 vs. control; p = 0.019 vs. MBZ 0.1 μM), confirming a strong dose-response. At 1 μM, MBZ induced a modest additional decrease in survival to approximately 65% (p = 0.001 vs. control), suggesting that cytotoxicity may plateau beyond the 0.5 μM threshold.

**Fig 1:**
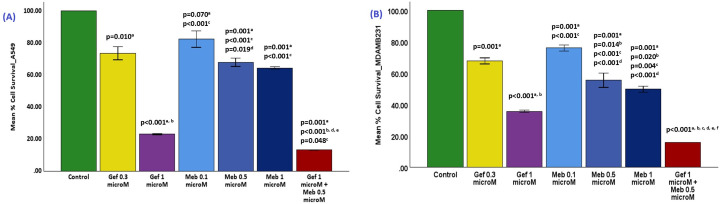
Effects of Gefitinib (Gef) and Mebendazole (Meb) on Cell Survival in A549 and MDA-MB-231 Cells. Cell survival was assessed following treatment with Gefitinib (Gef), Mebendazole (Meb), or their combination. Panel A: A549 lung adenocarcinoma cells; Panel B: MDA-MB-231 breast cancer cells. Data are expressed as percentage viability relative to untreated control (mean ± SD). Statistical comparisons: a vs. control; b vs. Gef 0.3 μM; c vs. Gef 1 μM; d vs. Meb 0.1 μM; e vs. Meb 0.5 μM; f vs. Meb 1 μM. The final bar shows Gef 1 μM + Meb 0.5 μM.

The combination treatment (gefitinib 1 µM + MBZ 0.5 µM) produced the most dramatic effect, reducing survival to ~8–10% (p = 0.001 vs control; p < 0.001 vs all monotherapies). This represents an additional >50% decrease in viability relative to the best single-agent treatment (gefitinib 1 µM), confirming strong synergism rather than additive toxicity (Fig 1A). These findings establish that gefitinib 1 µM alone has substantial cytotoxic activity, but its combination with MBZ 0.5 µM multiplies the effect, most likely via dual suppression of EGFR-dependent and cytoskeletal survival pathways.

### 3.2. Cell survival assays in MDA-MB-231 cells

In MDA-MB-231 triple-negative breast cancer (TNBC) cells, untreated controls maintained 100% viability. Gefitinib (0.3 µM) reduced survival to ~69% (p = 0.001), whereas gefitinib 1 µM markedly lowered survival to ~35% (p < 0.001 vs control and p < 0.001 vs gefitinib 0.3 µM), reflecting dose-intensified sensitivity (Fig 1B).

Mebendazole monotherapy showed a similar trend. At 0.1 µM, survival was ~ 84% (p = 0.001 vs control); at 0.5 µM, it declined to ~60% (p = 0.001 vs control; p = 0.014 vs gefitinib 0.3 µM; p < 0.001 vs MBZ 0.1 µM); and at 1 µM, viability was ~ 56% (p = 0.004 vs control).

Combination treatment (gefitinib 1 µM + MBZ 0.5 µM) caused a profound drop in survival to ~15% (p < 0.001 vs control and all monotherapies), producing an additional ~30–40% reduction relative to the best monotherapy (gefitinib 1 µM). The magnitude of this effect again confirms a synergistic interaction, likely due to simultaneous EGFR blockade and microtubule disruption. Overall, gefitinib 1 µM + MBZ 0.5 µM consistently yielded the strongest cytotoxicity across both NSCLC and TNBC models, establishing it as the optimal synergistic combination.

After establishing that the MBZ–GEF combination markedly reduced viability in both A549 and MDA-MB-231, we focused qRT-PCR analyses on A549 (NSCLC) cells, aligning with gefitinib’s approved clinical indication. The resulting transcriptional changes are therefore interpreted as most directly relevant to lung cancer, without extrapolation to TNBC. Gefitinib 1 µM monotherapy was excluded from qRT-PCR analyses because at this concentration, EGFR signaling is maximally inhibited, leading to widespread downstream transcriptional silencing that can obscure interaction-specific molecular responses. Such high-dose monotherapy produces a ceiling effect on EGFR-driven transcription, thereby diminishing the dynamic range necessary to detect synergism-dependent gene regulation. To preserve mechanistic resolution and distinguish cooperative signaling alterations rather than maximal pathway shutdown, a submaximal concentration of gefitinib (0.3 µM) and corresponding single-agent mebendazole doses were included. This approach ensured that observed differential expression profiles reflected genuine synergistic network modulation rather than nonspecific consequences of complete EGFR blockade.

### 3.3. Ras-related nuclear protein (RAN) gene expression analysis

In controls, RAN expression was set at a relative fold change of 1.0. Gefitinib (0.3 μM) significantly upregulated RAN to >2.2-fold (p < 0.001). This suggests that gefitinib, either via EGFR inhibition or compensatory pathways, may enhance transcription of RAN, a GTPase critical for nucleocytoplasmic transport and spindle assembly.

In contrast, MBZ consistently downregulated RAN. At 0.1 μM, expression dropped to ~0.5-fold (p < 0.001). At 0.5 μM, it fell further to ~0.4-fold (p < 0.001 vs. control; p = 0.002 vs. MBZ 0.1 μM). At 1 μM, RAN remained suppressed near 0.4-fold.

**Fig 2:**
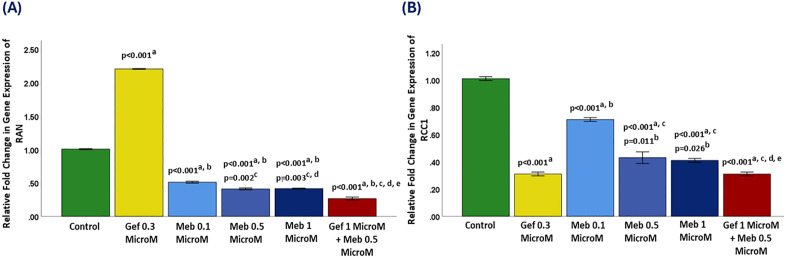
Relative expressions of RAN and RCC1 after treatment. Panel A: RAN; Panel B: RCC1. Groups include control, Gef 0.3 μM, Meb (0.1, 0.5, 1 μM), and Gef 1 μM + Meb 0.5 μM. Data are mean ± SD. Statistical comparisons: a vs. control; b vs. Gef 0.3 μM; c vs. Meb 0.1 μM; d vs. Meb 0.5 μM; e vs. Meb 1 μM.

The strongest effect came with combination treatment (gefitinib 1 μM + MBZ 0.5 μM), which reduced expression to ~0.3-fold (p < 0.001 vs. all groups; Fig 2A).

Given RAN’s central role in regulating cell division and genome stability, its marked downregulation by MBZ—and more so by the combination therapy- offers a compelling mechanistic explanation for the observed cytotoxicity. Inhibiting RAN function disrupts chromosomal alignment, spindle checkpoint fidelity, and proper mitotic progression, ultimately driving cells toward mitotic catastrophe and apoptosis. These findings position RAN as a critical downstream target of the MBZ–gefitinib combination and underscore the therapeutic potential of interfering with nuclear transport and mitotic control in highly proliferative cancer cells. Future studies should evaluate whether RAN suppression correlates with cell cycle arrest markers and mitotic failure *in vivo*, thereby validating its utility as a mechanistic biomarker and therapeutic node.

### 3.4. Regulator of chromosome condensation 1 (RCC1) gene expression analysis

In the untreated control group, RCC1 gene expression was established at a baseline relative fold change of 1.0. Gefitinib treatment at 0.3 μM led to a pronounced and statistically significant downregulation of RCC1 expression to approximately 0.3-fold (p < 0.001). This indicates a strong suppressive effect of gefitinib on RCC1, a pivotal guanine nucleotide exchange factor (GEF) for RAN that orchestrates chromatin condensation, nuclear transport, and mitotic progression.

Mebendazole (MBZ) showed dose-dependent modulation. At 0.1 μM, expression increased to ~0.7-fold (p < 0.001 vs. control), possibly reflecting feedback to mitotic stress. At 0.5 and 1 μM, expression dropped to ~0.4-fold (p < 0.001 vs. control; p = 0.011 and p = 0.026 vs. gefitinib).

The combination therapy (gefitinib 1 μM + MBZ 0.5 μM) elicited the most profound suppression of RCC1 expression, reducing it to approximately 0.3-fold, the lowest level observed across all conditions. This reduction was highly statistically significant compared to all other groups (p < 0.001), and notably matched the downregulation seen with gefitinib monotherapy, indicating a synergistic or convergent effect on RCC1 transcription (Fig 2B).

As RCC1 functions upstream of RAN in the RAN-GTPase axis—critical for nucleocytoplasmic trafficking, spindle formation, and chromosomal segregation—its simultaneous downregulation alongside RAN underscores a coordinated disruption of mitotic machinery by the combination therapy [50,51]. This dual suppression likely contributes to mitotic arrest, spindle dysfunction, and ultimately cell death, reinforcing the mechanistic basis for the potent cytotoxic effects observed in treated cancer cells. These findings highlight the RCC1–RAN pathway as a vulnerable target in cancer cell proliferation and suggest that its inhibition through combined EGFR blockade and microtubule disruption may represent a rational and effective therapeutic strategy. Further studies are warranted to explore the post-transcriptional regulation of RCC1 and its interaction with checkpoint signaling under combination treatment conditions.

### 3.5. Matrix metalloproteinase-2 (MMP2) gene expression analysis

In the untreated control group, MMP-2 gene expression was defined at a baseline relative fold change of 1.0. Treatment with gefitinib at 0.3 μM resulted in a modest but statistically significant reduction in MMP-2 expression to approximately 0.9-fold (p < 0.001), suggesting a slight inhibitory effect on this matrix-degrading enzyme, which plays a critical role in tumor invasion and metastasis.

Mebendazole (MBZ) showed a biphasic response. At 0.1 μM, expression increased to >1.8-fold (p < 0.001). At 0.5 μM, it returned to ~0.9-fold (p < 0.001 vs. control and MBZ 0.1 μM). At 1 μM, it dropped further to ~0.6-fold (p < 0.001), confirming a strong inhibitory effect at higher concentrations. This suggests that MBZ, at elevated doses, can robustly suppress genes involved in cancer cell invasion and matrix degradation.

**Fig 3:**
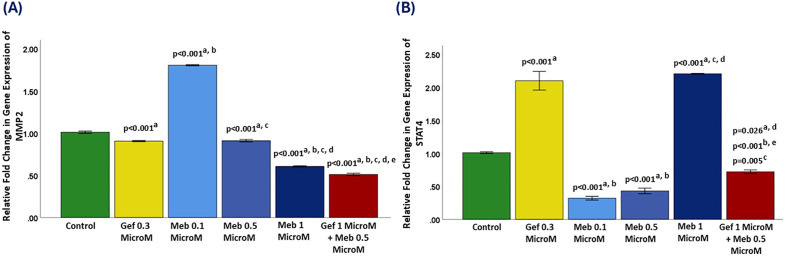
Relative expressions of MMP2 and STAT4 after treatment. Panel A: MMP2; Panel B: STAT4. Groups include control, Gef 0.3 μM, Meb (0.1, 0.5, 1 μM), and Gef 1 μM + Meb 0.5 μM. Data are mean ± SD. Statistical comparisons: a vs. control; b vs. Gef 0.3 μM; c vs. Meb 0.1 μM; d vs. Meb 0.5 μM; e vs. Meb 1 μM.

The combination treatment (gefitinib 1 μM + MBZ 0.5 μM) exhibited the most substantial downregulation of MMP-2, reducing expression to approximately 0.5-fold. This effect was highly significant compared to all other groups (p < 0.001), indicating a synergistic suppression of MMP-2 when both agents are administered concurrently (Fig 3A).

Given MMP-2’s well-established role in promoting tumor metastasis through extracellular matrix breakdown and angiogenesis, its downregulation by the combination therapy suggests a potential anti-metastatic benefit. These findings reinforce the therapeutic value of this dual-agent approach not only in inducing cytotoxicity but also in disrupting invasive behavior and metastatic potential in tumor cells.

### 3.6. Signal transducer and activator of transcription 4 (STAT4) gene expression analysis

STAT4 gene expression in the untreated control group was established at a baseline relative fold change of 1.0. Treatment with gefitinib at 0.3 μM significantly upregulated STAT4 expression to over 2.1-fold (p < 0.001), suggesting that EGFR inhibition may enhance signaling pathways linked to immune activation or cellular stress, as STAT4 is typically involved in Th1 immune responses and IFN-γ transcription.

In contrast, mebendazole (MBZ) at lower concentrations resulted in pronounced downregulation of STAT4. At 0.1 μM and 0.5 μM, MBZ suppressed STAT4 expression to approximately 0.3-fold and 0.4-fold, respectively (both p < 0.001 vs. control). These findings indicate that MBZ, at sub-micromolar levels, exerts a strong inhibitory effect on STAT4 expression, possibly by interfering with upstream cytokine signaling such as IL-12 or modulating transcriptional regulators linked to immune suppression. Interestingly, at 1 μM, MBZ significantly increased STAT4 expression to over 2.2-fold (p < 0.001 vs. control, p = 0.005 vs. MBZ 0.5 μM), mirroring the biphasic expression pattern observed for other cytokines in this study. This suggests that higher MBZ concentrations may activate immune-related transcriptional programs or stress-induced transcription factors contributing to STAT4 induction.

The combination treatment (gefitinib 1 μM + MBZ 0.5 μM) resulted in a moderate suppression of STAT4 expression to approximately 0.7-fold. This level was statistically significant compared to the control (p = 0.026), gefitinib (p < 0.001), and high-dose MBZ (p = 0.005), indicating that the dual therapy attenuated the pro-inflammatory STAT4 upregulation induced by either agent alone at higher doses (Fig 3B).

Given STAT4’s pivotal role in promoting Th1 polarization and enhancing cytotoxic immune responses, its modulation by the combination therapy suggests a controlled immunomodulatory effect. The partial downregulation may reflect a strategic dampening of excessive immune activation, potentially prevent immune exhaustion or chronic inflammation while maintaining antitumor efficacy. These findings underscore the complexity of immune gene regulation under combination therapy and highlight the importance of dose and context in shaping transcriptional outcomes. Further exploration of STAT4 protein activity and downstream signaling in immune-competent models would provide greater insight into its functional relevance in this therapeutic setting.

### 3.7. Interleukin-6 (IL-6) gene expression analysis

In the untreated control group, IL-6 gene expression was set as the baseline with a relative fold change of 1.0. Gefitinib treatment at 0.3 μM resulted in a significant upregulation of IL-6 expression, exceeding a 3.0-fold increase compared to the control (p < 0.001). This marked elevation suggests that while gefitinib effectively targets EGFR signaling, it may concurrently activate compensatory or stress-related pathways that drive IL-6 production—a cytokine known to support tumor growth, immune evasion, and therapeutic resistance.

**Fig 4:**
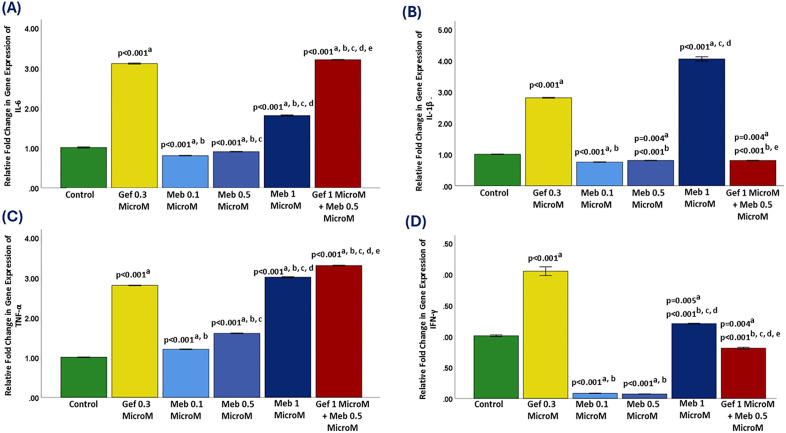
Relative expressions of pro-inflammatory cytokines (IL-6, IL-1β, TNF-α, IFN-γ). Panels: A, IL-6; B, IL-1β; C, TNF-α; D, IFN-γ. Groups include control, Gef 0.3 μM, Meb (0.1, 0.5, 1 μM), and Gef 1 μM + Meb 0.5 μM. Data are mean ± SD. Statistical comparisons: a vs. control; b vs. Gef 0.3 μM; c vs. Meb 0.1 μM; d vs. Meb 0.5 μM; e vs. Meb 1 μM.

In contrast, mebendazole (MBZ) monotherapy demonstrated a dose-dependent suppressive effect on IL-6 expression at lower concentrations. At 0.1 μM, MBZ significantly reduced IL-6 levels to approximately 0.8-fold (p < 0.001), and a similar downregulation was observed at 0.5 μM (approximately 0.9-fold; p < 0.001 vs. control). These results suggest that low-dose MBZ can attenuate IL-6 expression, potentially reducing inflammation-associated tumor progression. However, this was reversed at the highest MBZ concentration tested. At 1 μM, IL-6 expression was significantly upregulated to approximately 1.9-fold, a statistically robust increase compared to the control (p < 0.001), gefitinib (p < 0.001), and lower MBZ doses (p < 0.001 vs. 0.1 μM and 0.5 μM). This biphasic response—where low MBZ concentrations suppress IL-6 while higher doses stimulate its expression—mirrors the pattern previously observed for IL-1β and suggests a dose-dependent shift in the cellular stress response (Fig 4A).

Notably, the combination therapy (gefitinib 1 μM + MBZ 0.5 μM) resulted in an even greater increase in IL-6 expression, reaching approximately 3.2-fold. This was the highest expression level observed among all groups and was statistically significant compared to every other treatment (p < 0.001). The fact that IL-6 induction exceeded that of gefitinib alone highlights a potentially synergistic effect on inflammatory gene expression, despite the concurrent enhancement of cytotoxicity demonstrated in cell viability assays.

### 3.8. Interleukin-1 beta (IL-1β) gene expression analysis

IL-1β gene expression in the untreated control group was established as the baseline, with a relative fold change of 1.0. Treatment with gefitinib at 0.3 μM resulted in a significant upregulation of IL-1β expression, reaching approximately 2.9-fold (p < 0.001). This suggests that gefitinib monotherapy, despite its antiproliferative action, may simultaneously trigger inflammatory signaling pathways associated with IL-1β induction.

In contrast, mebendazole (MBZ) at lower concentrations exerted a suppressive effect on IL-1β expression. Treatment with MBZ at 0.1 μM significantly reduced IL-1β levels to approximately 0.8-fold (p < 0.001), and a similar downregulation was observed at 0.5 μM, with expression at approximately 0.9-fold (p = 0.004 vs. control; p < 0.001 vs. gefitinib). These results indicate that lower to moderate doses of MBZ can attenuate pro-inflammatory cytokine expression. Interestingly, a distinct biphasic pattern was observed with MBZ treatment. At 1 μM, IL-1β expression was markedly upregulated to over 4.0-fold—significantly higher than all other groups (p < 0.001 vs. control, MBZ 0.1 μM, and MBZ 0.5 μM). This suggests that while lower doses of MBZ may suppress IL-1β, higher concentrations may paradoxically induce inflammatory gene expression, possibly due to elevated cellular stress or compensatory responses.

The most notable effect was observed with the combination therapy (gefitinib 1 μM + MBZ 0.5 μM), which reduced IL-1β expression to approximately 0.8-fold, like the lowest MBZ dose (Fig 4B). This suppression was statistically significant compared to the control (p = 0.004), and highly significant versus both gefitinib monotherapy (p < 0.001) and high-dose MBZ (p < 0.001).

The combination thus effectively neutralized the IL-1β-inducing effects of each agent when used individually, restoring cytokine expression to near-baseline levels. Given IL-1β’s role as a potent pro-inflammatory cytokine and contributor to tumor-promoting inflammation, angiogenesis, and immune evasion, its suppression by the combination therapy suggests a favorable immunomodulatory effect. These findings indicate that dual targeting with MBZ and gefitinib not only enhances cytotoxicity but may also mitigate chronic inflammatory signaling within the tumor microenvironment—a key consideration for improving immune responsiveness and therapeutic outcomes.

### 3.9 Tumor necrosis factor-alpha (TNF-α) gene expression analysis

In the untreated control group, TNF-α gene expression was maintained at a baseline relative fold change of 1.0. Gefitinib at 0.3 μM significantly upregulated TNF-α expression to approximately 2.8-fold (p < 0.001), suggesting that EGFR inhibition may stimulate pro-inflammatory signaling cascades involving TNF-α. As a pleiotropic cytokine, TNF-α plays a dual role in cancer biology—capable of initiating both antitumor immunity and tumor-promoting inflammation depending on the cellular context.

Mebendazole (MBZ) treatment at lower concentrations demonstrated a more moderate influence on TNF-α expression. At 0.1 μM, MBZ induced a modest yet statistically significant increase in TNF-α to approximately 1.2-fold (p < 0.001), while 0.5 μM MBZ further elevated TNF-α to around 1.6-fold (p < 0.001 vs. control). Although these values reflect increased expression relative to baseline, they remained substantially lower than that observed with gefitinib, indicating a partial modulation of the inflammatory axis at these concentrations. In contrast, high-dose MBZ (1 μM) markedly upregulated TNF-α expression to approximately 3.0-fold, representing a significant increase compared to the control (p < 0.001), gefitinib (p < 0.001), and both lower MBZ doses (p < 0.001). This dose-dependent pattern aligns with significance seen for IL-1β and IL-6, suggesting a shared underlying mechanism of pro-inflammatory gene activation at elevated MBZ concentrations—potentially linked to microtubule disruption, oxidative stress, or mitotic stress responses.

The most pronounced elevation of TNF-α expression was observed in the combination therapy group (gefitinib 1 μM + MBZ 0.5 μM), which reached over 3.3-fold. This increase was statistically significant compared to all other groups (p < 0.001), surpassing the effects of either monotherapy (Fig 4C).

The additive or synergistic effect on TNF-α upregulation may indicate that the combination elicits a cellular stress response, which could contribute to immunogenic cell death or enhanced apoptotic signaling via TNFR1-associated pathways such as caspase-8 and JNK. These findings point to a complex immunomodulatory profile for the combination treatment. While elevated TNF-α is typically associated with inflammation, its context-dependent role in promoting tumor cell apoptosis may explain its concurrent upregulation alongside enhanced cytotoxicity in viability assays. Further mechanistic studies, particularly at the protein level and within *in vivo* systems, are warranted to delineate whether this TNF-α surge supports therapeutic efficacy or introduces inflammatory liabilities.

### 3.10. Interferon-gamma (IFN-γ) gene expression analysis

In the untreated control group, IFN-γ gene expression was set at a baseline relative fold change of 1.0. Treatment with gefitinib at 0.3 μM led to a significant upregulation of IFN-γ, reaching approximately 2.1-fold (p < 0.001). This suggests that gefitinib may trigger immune-related or stress-associated signaling pathways that enhance IFN-γ transcription, a cytokine known for its immunostimulatory and antiproliferative effects.

In contrast, mebendazole (MBZ) at lower concentrations resulted in marked suppression of IFN-γ expression. Both 0.1 μM and 0.5 μM MBZ treatments significantly downregulated IFN-γ to approximately 0.1-fold (p < 0.001 vs. control), indicating a strong inhibitory effect. This downregulation suggests that MBZ, at these doses, may suppress pathways associated with interferon signaling or reduce IFN-γ production as part of its stress response modulation. Interestingly, at a higher concentration of 1 μM, MBZ significantly increased IFN-γ expression to approximately 1.2-fold (p = 0.005 vs. control; p < 0.001 vs. lower MBZ doses and gefitinib). This biphasic response—strong suppression at low doses and stimulation at high doses—parallels expression observed for other cytokines such as IL-1β and IL-6, suggesting that MBZ elicits dose-dependent modulation of immune gene expression.

The combination treatment (gefitinib 1 μM + MBZ 0.5 μM) resulted in a reduction of IFN-γ expression to approximately 0.8-fold (Fig 4D). This suppression was statistically significant when compared to the control (p = 0.004), and highly significant relative to gefitinib monotherapy (p < 0.001), MBZ 0.1 μM and 0.5 μM (p < 0.001), and MBZ 1 μM (p < 0.001).

These results indicate that the combination therapy not only neutralized the gefitinib-induced IFN-γ elevation but also mitigated the immune activation observed at high-dose MBZ. Given the dual role of IFN-γ—promoting antitumor immunity in acute settings but contributing to immune exhaustion and chronic inflammation under persistent expression—its downregulation by the combination may offer therapeutic advantages. It could minimize potential immune suppression or reduce the risk of tumor adaptation to sustained interferon signaling. Overall, these findings suggest that while gefitinib alone activates IFN-γ transcription and MBZ exerts a dose-dependent modulatory effect, the combination therapy tempers this response. This may contribute to a more balanced and immune-permissive tumor microenvironment, potentially enhancing compatibility with future immunotherapeutic strategies.

## 4. Discussion

This *in vitro* investigation examined the therapeutic synergy between mebendazole (MBZ) and gefitinib (GEF) in two of the most aggressive and therapy-resistant cancer cell lines: A549 (NSCLC) and MDA-MB-231 (TNBC). These models are clinically relevant due to their inherent resistance mechanisms, including EGFR pathway alterations, epithelial–mesenchymal transition (EMT), immune escape, and enhanced drug efflux [52,53]. Our data provide compelling evidence that the combination of GEF 1 µM and MBZ 0.5 µM elicits superior cytotoxic, transcriptional, and immunomodulatory effects compared to either agent alone. Our findings demonstrate that MBZ and GEF exert strong combined cytotoxic effects *in vitro*; however, we emphasize that these results provide a mechanistic hypothesis for future testing in animal models and, eventually, clinical studies. Specifically, claims about overcoming resistance and immuno-oncology applications are presented here as plausible directions for future research rather than definitive conclusions.

The selection of gefitinib (GEF) 1 µM for combination studies was guided by its pronounced dose-dependent cytotoxicity in both A549 and MDA-MB-231 cell lines, where residual cell viability ranged between ~20–35% relative to untreated controls. When co-administered with mebendazole (MBZ) 0.5 µM, viability was further reduced to below 15%, representing the most significant synergistic response (p < 0.001). This combination was therefore chosen for downstream mechanistic qRT-PCR profiling to elucidate the molecular correlations of synergy. However, GEF 1 µM monotherapy was deliberately excluded from gene-expression analysis because, at this concentration, EGFR signaling is already maximally suppressed, producing extensive downstream transcriptional quiescence. Inclusion of such a high-dose monotherapy would confound the interpretation of differential gene expression by masking synergism-specific regulatory interactions under conditions of complete pathway saturation. Instead, a submaximal gefitinib dose (0.3 µM) and corresponding single-agent MBZ exposures were analyzed, as these conditions maintain partial EGFR activity and a dynamic transcriptional range, allowing the identification of cooperative signaling perturbations and true synergy-driven transcriptional programs rather than non-specific consequences of full EGFR blockade.

Our decision to restrict downstream qRT-PCR to A549 was deliberate and grounded in translational relevance: gefitinib is an EGFR tyrosine-kinase inhibitor used primarily in NSCLC, whereas data supporting routine use in TNBC are limited and context-dependent. Focusing gene-level interrogation on the lung cancer model (A549) allowed us to (i) align molecular readouts with the drug’s clinical indication, (ii) concentrate on EGFR-axis and cytoskeletal pathways central to our priori hypothesis, and (iii) limit multiple comparisons that inflate false-positive rates when profiling across divergent disease contexts.

Gefitinib, a first-generation EGFR tyrosine kinase inhibitor (TKI), inhibits receptor autophosphorylation and downstream pathways such as PI3K/AKT/mTOR and MAPK/ERK [54]. Despite its success in EGFR-mutant tumors, resistance mechanisms—like MET amplification [54,55] and KRAS mutations—limit its standalone efficacy [56]. MBZ, traditionally an anthelmintic agent, disrupts microtubule polymerization via β-tubulin binding, inducing mitotic catastrophe. Its anticancer repurposing is supported by its low toxicity, ability to cross the blood-brain barrier, and capacity to generate reactive oxygen species (ROS), activate p53, and arrest the cell cycle at G2/M [12].

The combination of MBZ (0.5 µM) and GEF (1 µM) resulted in marked cytotoxicity in both A549 and MDA-MB-231 cells—exceeding the effects of monotherapy. This synergy appears to result from converging mechanisms: GEF-mediated inhibition of proliferation sensitizes cells to MBZ-induced mitotic stress, while MBZ amplifies GEF’s anti-survival effects via ROS generation and mitochondrial disruption. The observed cytotoxicity may involve activation of intrinsic apoptotic pathways, including caspase-3, BAX, cytochrome c release, and PARP cleavage, but this remains to be directly confirmed. In A549 cells with high EGFR expression, this combination effectively shuts down proliferative signaling while enforcing cell cycle arrest. In TNBC cells, where EGFR acts as a compensatory growth signal in the absence of ER, PR, and HER2, the dual inhibition significantly impairs key survival pathways [57]. This may reduce stemness, migration, and EMT-related phenotypes that are central to the aggressive nature of MDA-MB-231 cells. The combination thus targets complementary vulnerabilities, providing a strong rationale for its use in EGFR-independent cancers.

Gene expression profiling revealed a distinct immunological signature induced by the combination therapy. Notably, IL-1β and IFN-γ were significantly downregulated. IL-1β is central to inflammasome activation and tumor-promoting inflammation via NLRP3 and NF-κB signaling. Its suppression may reflect diminished inflammasome activation or reduced ROS levels [58–63]. IFN-γ, despite its antitumor potential, can also induce immune exhaustion in chronic inflammation; its reduction may therefore be beneficial in reshaping the tumor microenvironment [64]. In contrast, IL-6 and TNF-α were upregulated following combination treatment. IL-6, though associated with tumor progression via STAT3 activation, can initiate negative feedback through SOCS3 or STAT3 degradation during acute stress [65,66]. TNF-α could contribute to immunogenic cell death (ICD) by influencing apoptotic pathways such as caspase-8 and JNK, though this possibility requires further investigation. These cytokine shifts suggest a transient pro-inflammatory response capable of recruiting immune cells and promoting dendritic cell activation through DAMP release (e.g., HMGB1, ATP). Importantly, these transcriptional changes represent early responses; later stages may involve translational repression, mRNA decay, and protein degradation, mitigating potential long-term pro-tumorigenic effects. This dual modulation of inflammatory mediators—suppressing chronic inflammation (IL-1β, IFN-γ) while inducing acute immune activation (IL-6, TNF-α)—may reprogram the tumor microenvironment toward an immune-permissive state. As such, this combination could serve as a priming strategy for immune checkpoint inhibitors, particularly in immune-cold tumors. The biphasic responses observed for IL-6, IL-1β, and IFN-γ suggest a dose-dependent switch in cellular behavior. At low doses, MBZ suppressed cytokine expression, potentially reflecting reduced basal inflammatory signaling. At higher doses, MBZ induced cytokine upregulation, which may reflect heightened cellular stress, ROS generation, or activation of compensatory survival pathways. The combination with GEF further amplified these responses. Importantly, these results were obtained *in vitro* without immune cells; thus, interpretation is limited. Future time-course experiments and studies in immune-competent models will be essential to determine whether these biphasic patterns represent transient stress responses, pro-inflammatory danger signals, or chronic tumor-promoting inflammation.

Further transcriptional insights reveal that combination therapy significantly impacts genes involved in mitotic integrity and immune regulation. Downregulation of RAN and RCC1 suggests disruption of nucleocytoplasmic transport and mitotic spindle formation, reinforcing the mitotic stress induced by MBZ. These changes impair chromosome segregation and may trigger cell death via mitotic catastrophe. Downregulation of RAN and RCC1 observed in this study may reflect either (i) a primary effect of MBZ on microtubule and nuclear transport machinery and/or GEF on EGFR signaling, or (ii) a secondary downstream consequence of cell death pathways. Because mRNA levels alone cannot distinguish these possibilities, future studies should incorporate protein-level validation, siRNA knockdown experiments, and mechanistic assays to confirm whether suppression of the RAN–RCC1 axis is causative or secondary. STAT4 upregulation, typically linked to Th1 polarization and IFN-γ production, suggests a context-dependent role in enhancing anti-tumor immunity. Meanwhile, MMP-2, involved in ECM remodeling and metastasis, showed modulation likely secondary to cytoskeletal destabilization or SASP-related signaling. It is important to interpret these results considering mRNA–protein correlations, which are not always direct due to translational control and post-translational modifications. Future work involving proteomics, cytokine quantification, and metabolomics is necessary to validate and extend these findings. From a clinical perspective, this dual-drug strategy addresses both oncogenic signaling and cytoskeletal resilience—two core cancer hallmarks. It may permit dose reductions of each agent, minimizing adverse effects and mitigating resistance development. Notably, MBZ and GEF have favorable safety profiles and are already approved for clinical use, facilitating rapid translational application.

In comparative terms, gefitinib alone at 0.3 µM only partially inhibited EGFR signaling, while MBZ at 1 µM disrupted mitosis but lacked upstream suppression of proliferative signaling. The combination at lower doses produced superior results by targeting both axes simultaneously, supporting a rationale for combination regimens in solid tumors. Mechanistically, this regimen may engage cellular stress pathways such as ATM/ATR, CHK1/CHK2, and the unfolded protein response (UPR), although this requires further validation. The resulting translational arrest, mRNA decay, and autophagy further contribute to cytotoxicity. The upregulation of STAT4 and TNF-α is particularly relevant in NSCLC, where immune resistance via PD-L1 overexpression is prevalent. By potentially reversing immune escape and restoring antigenicity, this combination may augment the efficacy of PD-1/PD-L1 or CTLA-4 blockade therapies. While multiple cytokine and gene expression changes reached statistical significance in our qRT-PCR analyses, it is important to distinguish between statistical and biological significance. For instance, IL-6 and STAT4 exhibited only modest fold increases in the range of 1.2–1.5, which, although reproducible across replicates, may reflect subtle transcriptional modulation with uncertain downstream impact. In contrast, MMP-2 demonstrated a more robust increase exceeding two-fold, which is more likely to represent meaningful transcriptional reprogramming with functional consequences for tumor invasiveness and extracellular matrix remodeling. This pattern illustrates that not all statistically validated changes are equally relevant biologically.

This study offers several notable strengths. Its multi-parametric design, which combines cell viability assays with transcriptional profiling, provides a comprehensive evaluation of the effects of the MBZ–GEF combination. The use of clinically relevant cancer models adds translational value, while the integration of functional and molecular endpoints allows for a more nuanced understanding of how cytoskeletal disruption and EGFR inhibition may interact to impair cancer cell survival. By focusing qRT-PCR analyses on A549 cells, we aligned molecular interrogation with the clinical setting in which gefitinib is most relevant, thereby increasing translational specificity to NSCLC. At the same time, this choice limits the generalizability of gene-level conclusions to TNBC, despite the demonstration of cytotoxic effects in both models. Extending transcriptomic and protein-level validation to MDA-MB-231 and other TNBC lines will therefore be an important priority for future work.

This study has some limitations that provide opportunities for future research. We focused on two cancer cell lines (A549 and MDA-MB-231) and did not include normal control cells, which may slightly limit generalizability. Our experiments measured mRNA expression without protein-level confirmation, and while transcript–protein correlations are not always perfectly aligned, the findings still provide valuable mechanistic insights. Apoptosis and cell cycle assays were not included, leaving the detailed mechanisms of cell death to be further explored. The biphasic cytokine responses observed may reflect temporal dynamics not captured in our static assays, suggesting that time-course studies would be informative. Finally, as an *in vitro* study, *in vivo* validation is needed to confirm translational relevance. Addressing these points in future investigations, including protein-level analyses, apoptosis and cell cycle studies, siRNA knockdown experiments, and validation in xenograft or patient-derived models—will further strengthen and extend the impact of these findings.

Taken together, our results demonstrate that the combination of mebendazole and gefitinib produces a multifaceted anticancer response, characterized by mitotic disruption, EGFR pathway inhibition, immune-related transcriptional modulation, and evidence of synergistic cytotoxicity. While these findings strongly suggest therapeutic potential *in vitro*, their true clinical relevance will depend on validation *in vivo*, particularly in patient-derived xenografts and immune-competent models that can capture both tumor biology and host responses. Such studies will be critical to determine whether the MBZ–GEF combination can realistically overcome therapeutic resistance and provide a rational foundation for future integration into immuno-oncology frameworks.

## 5. Conclusions

This study provides strong preclinical evidence that MBZ and GEF synergistically reduce viability in both NSCLC and TNBC cell lines and modulate gene expression in NSCLC cell lines. Importantly, these data are preliminary and must be interpreted within the limitations of an *in vitro* system. We propose that these findings form a hypothesis-generating platform for future studies, including mechanistic protein-level assays, apoptosis/cell cycle analysis, and *in vivo* validation, to confirm whether this dual-targeting strategy can realistically contribute to overcoming resistance in aggressive cancers.

## Supporting information

S1 File Minimal data set. (XLSX)
